# The socialization effect on decision making in the Prisoner's Dilemma game: An eye-tracking study

**DOI:** 10.1371/journal.pone.0175492

**Published:** 2017-04-10

**Authors:** Anastasia G. Peshkovskaya, Tatiana S. Babkina, Mikhail G. Myagkov, Ivan A. Kulikov, Ksenia V. Ekshova, Kyle Harriff

**Affiliations:** 1 Laboratory of Experimental Methods in Cognitive and Social Sciences, Tomsk State University, Tomsk, Tomsk Region, Russian Federation; 2 Mental Health Research Institute, Tomsk National Research Medical Center, Russian Academy of Sciences, Tomsk, Tomsk Region, Russian Federation; 3 Center for Design, Manufacturing and Materials, Skolkovo Institute of Science and Technology, Moscow, Russian Federation; 4 Department of Political Science, University of Oregon, Eugene, Oregon, United States; Tianjin University of Technology, CHINA

## Abstract

We used a mobile eye-tracking system (in the form of glasses) to study the characteristics of visual perception in decision making in the Prisoner's Dilemma game. In each experiment, one of the 12 participants was equipped with eye-tracking glasses. The experiment was conducted in three stages: an anonymous Individual Game stage against a randomly chosen partner (one of the 12 other participants of the experiment); a Socialization stage, in which the participants were divided into two groups; and a Group Game stage, in which the participants played with partners in the groups. After each round, the respondent received information about his or her personal score in the last round and the overall winner of the game at the moment. The study proves that eye-tracking systems can be used for studying the process of decision making and forecasting. The total viewing time and the time of fixation on areas corresponding to noncooperative decisions is related to the participants’ overall level of cooperation. The increase in the total viewing time and the time of fixation on the areas of noncooperative choice is due to a preference for noncooperative decisions and a decrease in the overall level of cooperation. The number of fixations on the group attributes is associated with group identity, but does not necessarily lead to cooperative behavior.

## Introduction

The eyes are part of a synergetic system that reflects brain activity. The oculomotor activity of the eyes is a correlate of certain brain processes [1]. Eye-tracking offers significant opportunities for the study of decision making because it allows us to access the hidden internal forms of activity that we normally cannot observe and allows us to examine the both the results and the procedural characteristics of the decision making process. Recent works have investigated cooperation in evolutionary social dilemmas set factors that affect cooperation among agents, such as probabilistic participation and inactivity [2], reputation assortment [3], and coupling of utility assessment [4]. Studies on network structures are focused on such characteristics as the synchronization behavior of networks [5], attack vulnerability [6], controllability and network size [7], neighborhood size [8], and heterogeneous coupling between interdependent lattices [9], which are important for consideration in the complete description of real-world complex systems. These works provide significant advancement of our understanding of cooperation. At the same time, eye-tracking allows us to build behavioral models that consider the cognitive component, to make them more accurate.

Researchers in the field of experimental economics [10–13] apply eye-tracking methods to explore the strategic component of the behavior of the players, as well as various individual psychological characteristics that lead to certain decisions, rather than the output feedback of the results of each round. Studies that exclude feedback show that two decision making strategies can be identified that correspond to different levels of a level-k model [11]. In [14] it was noted that some results of applying eye-tracking methods in one-shot games could not be fully explained by level-k models (the models are described in [15–18] or cognitive hierarchy [19,20]). In [14] potential explanations for those results were shown in diffusal drift models, which allow one to analyze procedural data such as decision making time and eye movement characteristics. The general thrust of these studies is to explore the possibility of forecasting a player’s decision by tracking his or her eye movements, followed by verification and generalization of the results in various spheres of human interaction. However, the exclusion of output results makes researchers work exclusively with the pure strategic behavior of participants when they take into account their opponent's action only on the basis of their forecasts, while economic agents whose behavior is modeled in such experiments see the consequences of their behavior. Therefore, we believe it is important to include research feedback, otherwise the results obtained in laboratory experiments may not be able to interpret the real processes that they are intended to explain.

Today all experimental economics studies are conducted in computer classes. Our study of the characteristics of eye movements in the decision making process was also carried out in a computer class. The Prisoner's Dilemma game has been used as a research tool [21–24].

This study is part of a series of experiments whose main is to study the impact of socialization and social interaction on cooperation. It is known that people, being social animals, try to cooperate with each other because our survival depends on common goals and interactions within the group [25,26]. It was shown experimentally that a person within the group seeks cooperation and reciprocity, thereby improving his or her reputation [27–31]. Prosocial behavior can be generated through social interaction between members of the group, accompanied by identification with the group, which leads to the appearance of added value of collective interaction, as well as a sense of in-group favoritism and the positive emotions of being in a particular group [32,33]. Thus, sociality becomes a mechanism for boosting cooperation. Sociality serves as one more component in the utility function, responsible for the group result and group cohesion, and comes from a sense of social identity [34].

In the experiment, sociality is formed through social interaction or socialization, that is, through the familiarization of participants and their division into groups. This laboratory model combines the classic social psychology minimal group paradigm with group manipulations that cause a sense of social attachment [35,36]. The use of this model in the experiments revealed that socialization leads to higher levels of cooperation and its persistence in participants within social groups, compared to the initial level of cooperation in a random group of strangers [37].

In a series of experiments conducted at the Moscow Institute of Physics and Technology and Tomsk State University, the effect of socialization on cooperation was studied. The mobile eye-tracking system was used in nine experiments to find answers to the following questions:

1. How are decision making process data, obtained via a mobile eye-tracking system related to the level of cooperation?
2. Are there any differences between visual perception features before socialization and after it?
3. Which areas of a payoff matrix show the greatest difference in visual perception before and after socialization?

## Materials and methods

### Participants

Nine experiments were conducted. Twelve participants took part in each experiment. One participant of the 12 in each experiment was equipped with the mobile eye-tracking system. The sample for eye-movement recording (n = 9) included five women and four men aged from 20 to 40 years old (M = 23.7, SD = 6.2). All the participants were recruited on a voluntary basis through the social network VKontakte (vk.com). The study procedures involving human participants were approved by Tomsk State University Human Subjects Committee. Written informed consents were obtained from participants. Experimental data are readily available on Harvard Dataverse: https://goo.gl/0hDljy.

### Design and procedures

All the participants were invited to a computer class where they completed the participant’s consent form. One participant of 12 was randomly chosen out of the group who was then equipped with the mobile eye-tracking system Eye Tracking Glasses v.1.8. by SensoMotoric Instruments GmbH (hereinafter ETG). Following a brief description of the research procedures, the participants received written and verbal instructions for the Prisoner's Dilemma game.

#### Stage 1. Individual game

**Fig 1:**
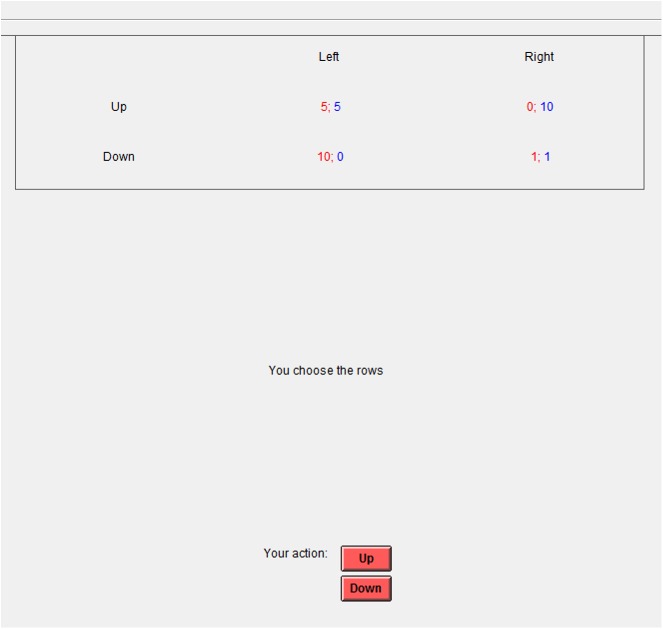
Example of stimulus material during the Individual Game. Screenshot at the stage of the Individual Game.

The participant with ETG did a 1-point calibration accuracy test. Then, all 12 participants proceeded to the Prisoner's Dilemma game (11 rounds). A specialized tool to design and carry out group experiments in experimental economics, z-Tree developed at the University of Zurich, was used [38] (Fig 1). The interface design for the participant with ETG was identical to the interface design of the other participants, and was used in all experiments.

The participants were able to move to the next round only after all 12 participants made their choices. No one knew who their opponents were and in each round the pairs of participants changed randomly. After each round, the result of the round and the overall result for the current point in the game were displayed on the monitor. After the game was completed, calibration verification took place and the participant removed the ETG.

#### Stage 2. Socialization

Social communication between the participants was built by a snowball exercise (each participant should remember the names of the other participants and their descriptions of themselves using a word beginning with the first letter of their name) and telling a brief story about themselves. Next, two captains were voluntarily selected and all the participants were divided into two groups (six members each). The participants were asked to find 3–5 characteristics common to all members of the group and choose a name for their group.

#### Stage 3. Group game

After the Socialization stage, the participants took their seats at the computers. They were instructed that they would be asked to play the Prisoner's Dilemma game again, but this time their partner would be a random member from the newly formed group of six people. The participant, who during the Individual Game stage was equipped with ETG, put on the ETG and completed the calibration accuracy test once again. Then all the participants proceeded to the Prisoner's Dilemma game, which consisted of 15 rounds. After each round, the participants could see the result of each round and the total personal result for the game at the moment, as well as the overall results for the two groups on their monitors.

After the Group Game stage the participants were tested to determine the index of group identity according to the Fishbach-Ellemers scale [39]. They also reported on the moves they used, as well as on those moments in the game that caused any experiences or thoughts.

The roles (who would select rows or columns) during the Individual Game and Group Game stages were assigned randomly so that each participant played approximately the same number of rounds in each role (6 and 5 before Socialization, 8 and 7 after Socialization).

**Fig 2:**
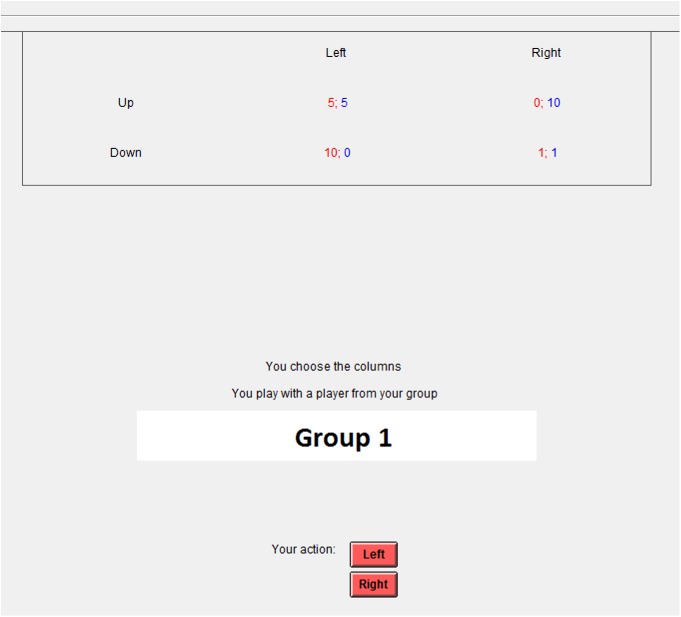
Example of stimulus material during the Group Game. Screenshot at the stage of the Group Game.

The gains matrix content was unchanging. However, at the Group Game stage, group names proposed by the participants were added to the screens (Fig 2).

Semantic Gaze Mapping, included in the SensoMotoric Instruments BeGaze, was used for aggregating each participant’s results on the stimulus matrix. Semantic Gaze Mapping allows us to determine the points of a subject gaze fixation on a stimulus for the selected trials, if the eye movements were recorded with the help of ETG. Further quantitative indicators were exported from BeGaze and statistically processed by StatSoft Statistica v. 10. Parameters of oculomotor activity in one round of the game were taken as the unit of analysis.

## Results and discussion

Result 1. The decision making process before Socialization is accompanied by greater Dwell Time and longer Fixation Time on the areas of noncooperative choices.

**Fig 3:**
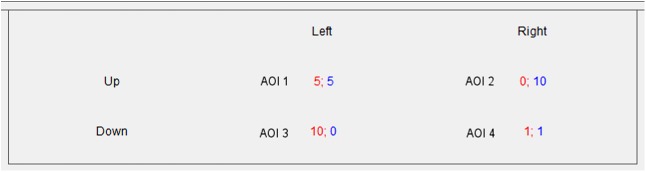
Selected areas of interest (AOI). The illustration represents the payoff matrix of the Prisoner's Dilemma. Every cell is the area of interest (AOI).

The cells of the Prisoner's Dilemma payoff matrix were selected as areas of interest (AOI) to compare the parameters of oculomotor activity during the stages of the Individual Game (before Socialization) and the Group Game (after Socialization) (Fig 3).

Before socialization, longer Dwell Time (Dwell Time, %) and longer Fixation Time (Fixation Time, %) was registered for AOI 3 (F 4.11, p = 0.008) and AOI 4 (F 4.0, p = 0.009) (Table 1).

**Table 1:** Dwell Time and Fixation Time before the Socialization stage.

	Dwell Time, %		Fixation Time, %	
	M	SD	M	SD
AOI 1	4.34	4.64	4.02	4.19
AOI 2	3.99	5.22	3.71	4.86
AOI 3	8.62	6.73	7.71	5.80
AOI 4	6.34	5.63	5.74	4.97

Dwell Time (Dwell Time, %) and Fixation Time (Fixation Time, %) before the Socialization stage in the different areas of interest (AOI).

AOI 3 in the case of a choice between the rows corresponds to the cell of noncooperative choice (defect), where the gain is 10 points at 0 points for the partner. For the participant, choosing columns AOI 3 corresponds to the cell of a choice to cooperate and a gain of 0 at 10 for the partner.

AOI 4 corresponds to a choice to defect for participants regardless of their role (whether they choose a row or column) and is equal to the minimum outcome with a gain of 1 point for each participant.

Moreover, there is no significant difference between decisions to defect and to cooperate (p = 0.092). According to the descriptive data on the frequencies, participants preferred to defect in 77.14% of rounds, if they selected rows and in 56.1% of rounds if they selected columns.

In general, the Individual stage before Socialization with greater Dwell Time and longer Fixation Time on the areas of defections (AOI 4 is always defection, AOI 3 is also defection, for the choice between rows, but not always for the choice between columns) is accompanied by a low level of cooperation—28.3% on average (S1 Table).

No differences in the parameters of oculomotor activity in areas of interest were found after Socialization (Wilks lambda 0.91, F 1.12, p = 0.339). Perception of gains cells in the payoff matrix after Socialization occurred with the same Dwell Time, Fixation Time, and number of gaze returns to all AOI.

Thus, after Socialization no preferred payoff matrix cells according to eye movement parameters were identified. The average percentage of cooperation of participants at this point was 57.8% (S2 Table), and it was higher than before the Socialization stage.

Result 2. The increase in Dwell Time and Fixation Time is related to a preference for defection and a decrease in the overall level of cooperation.

The majority of participants showed high cooperation after the Socialization stage, except for two participants, in whom reduction in the percentage of cooperation choices from 36% to 0% and from 45% to 27% (S1 Table, S2 Table) was observed, which raised the question of the existence of possible differences in oculomotor activity settings based on an increasing or decreasing level of cooperation. We combined data on the eye movements of participants that showed reduction in cooperation (Defectors) and compared them with data on those participants whose cooperation increased after Socialization (Cooperators).

ANOVA analysis revealed significant differences in the parameters of eye movements of the participants with regard to cooperation before (Individual Game) and after Socialization (Group Game) (Wilks lambda 0.84, F = 2.47, p = 0.039).

**Fig 4:**
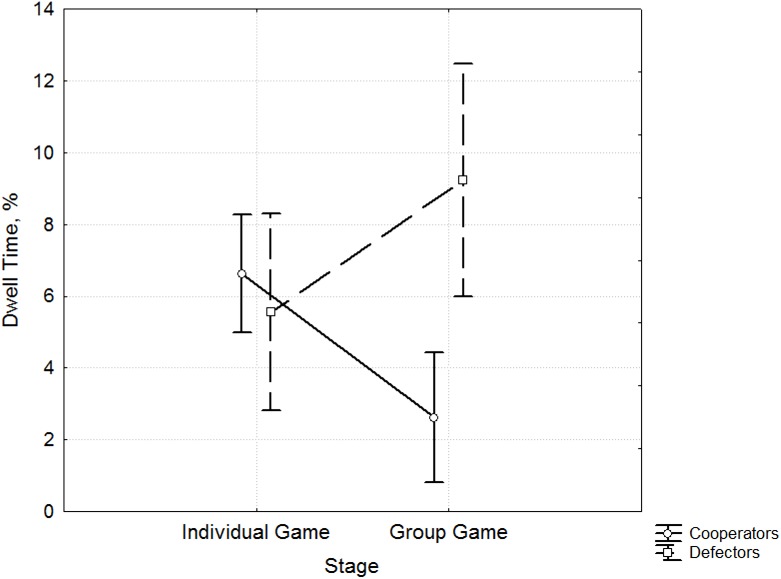
Differences in Dwell Time (%) for AOI 4. The differences in Dwell Time (y-axis) between Cooperators (blue) and Defectors (red) (x-axis) according to the behavior data from the Prisoner’s Dilemma game during the Individual Game and Group Game stages.

The Dwell Time for AOI 4 (S3 Table) corresponding to noncooperation decisions (defects) at the stage of the Group Game was greater among Defectors, the participants whose level of cooperation fell after socialization (F 9.71, p = 0.003) (Fig 4). Participants with an increased level of cooperation showed a shorter Dwell Time for AOI 4.

**Fig 5:**
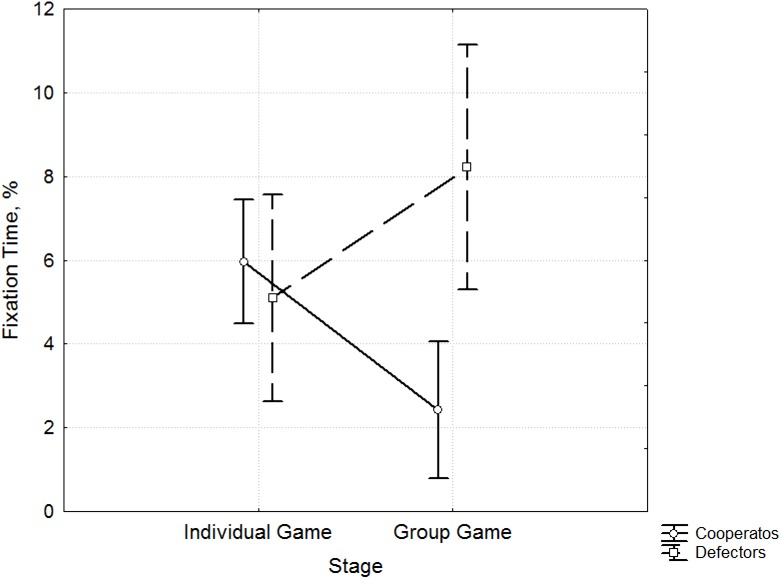
Differences in Fixation Time (%) for AOI 4. The differences in Fixation Time (y-axis) between Cooperators (blue) and Defectors (red) (x-axis) according to the behavior data from the Prisoner’s Dilemma game during the Individual Game and Group Game stages.

The Fixation Time for AOI 4 (S4 Table) is also significantly shorter in participants whose cooperation level increased during the Group Game stage (F 8.96, p = 0.004) (Fig 5).

Result 3. The number of gaze fixations in the area of the group name is higher in participants with a strong group identity.

During the Socialization stage the participants were divided into two groups. Each group consisted of six people; one of them was equipped with ETG. The participants in both groups were asked to find common characteristics for all their group members, as well as to come up with a name for their group, which then appeared on the screen during the Prisoner's Dilemma in the Group Game stage.

**Fig 6:**
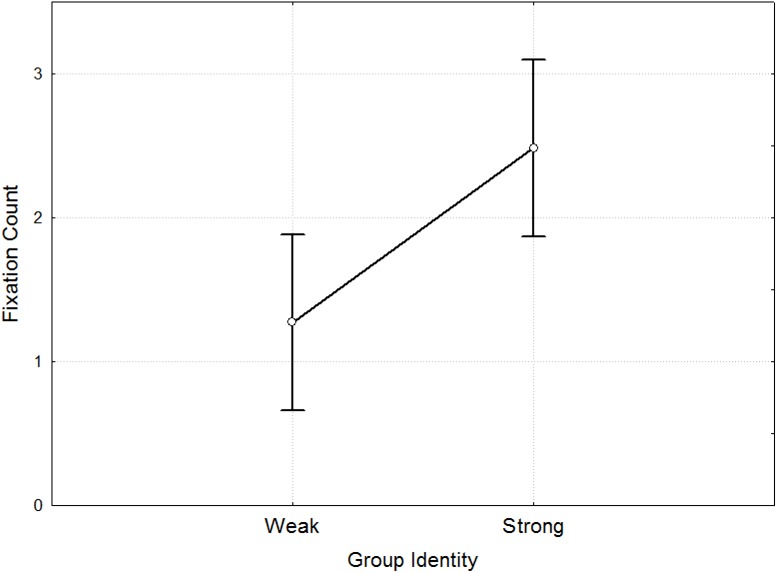
Differences in Fixation Frequency on group name in participants with a weak and strong group identity. The differences in Fixation Count (y-axis) between participants with weak and strong group identity (x-axis).

After the Group Game stage, the index of group identity was measured on the Fishbach–Ellemers scale [39], according to which each participant evaluated his or her relationship with the group formed at the Socialization stage from 1 (weak) to 7 (strong) points. A border result was 4.5 points, dividing the participants with a weak and a strong group identity (S5 Table). We found that the number of fixations on the name of the group was higher in participants with a strong group identity (F = 7.81, p = 0.007) (S6 Table). The number of gaze fixations on the group name in participants with a weak group identity was 1.27 (Fig 6), while in participants with a strong group identity it was 2.49.

However, no significant differences were found in the parameters of eye movements for the area with the name of the group in connection with the decisions taken by participants (F 0.01, p = 0.905). The average number of gaze fixations on the name of the group in Cooperators with a strong group identity was 2.4; in the case of Defector participants it was 2.6. The number of fixations on the name of the group in participants with a weak group identity was 1.2 for a cooperation decision and 1.3 for a defection decision. Thus, a greater number of gaze fixations on the name of the group is related to a strong group identity but is not due to the nature of a decision (preference for a “defect” or “cooperate” decision).

Result 4. Socialization influenced the nature of stimuli perception in the Prisoner's Dilemma game. The decision making process after socialization is accompanied by an increase in Fixation Frequency and reduction in Fixation Duration, as well as greater Saccade Frequency and shorter Scanpath Length.

To compare the general characteristics of oculomotor activity during the stages before and after socialization, mean and frequency variables and one generalizing variable were used:

1. Visual Intake Frequency (count/s) is the number of gaze fixations per second;
2. Visual Intake Duration Average (ms) is the average fixation duration;
3. Saccade Frequency (count/s) is the number of saccades per second;
4. Scanpath Length (px) is the total length of all saccades in gaze trajectory.

ANOVA analysis showed significant differences between stages before socialization (Individual Game) and after socialization (Group Game) (Wilks Lambda = 0.83496, F (6. 199) = 6.5559, p <0.000). The images below show the mean values and 95% confidence intervals.

**Fig 7:**
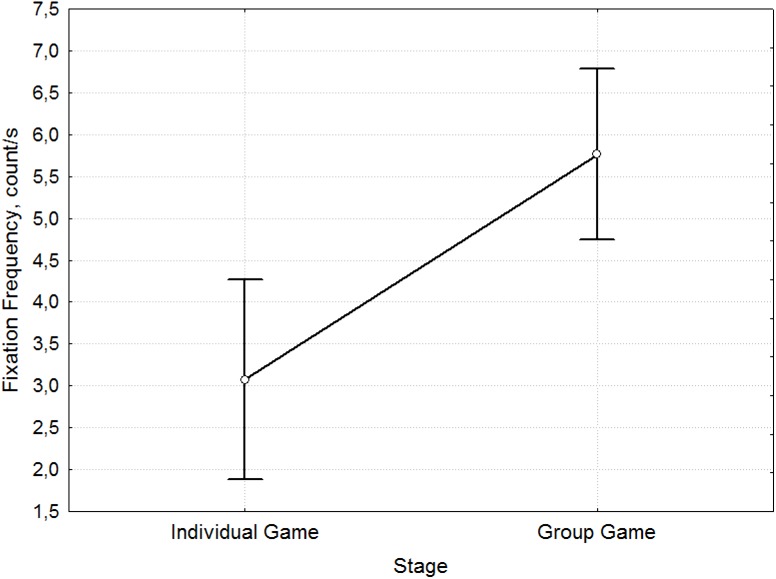
Mean comparison of Fixation Frequency for the stages before and after Socialization. The differences in Fixation Frequency [count/s] (y-axis) between the Individual Game and Group Game stages (x-axis).

**Fig 8:**
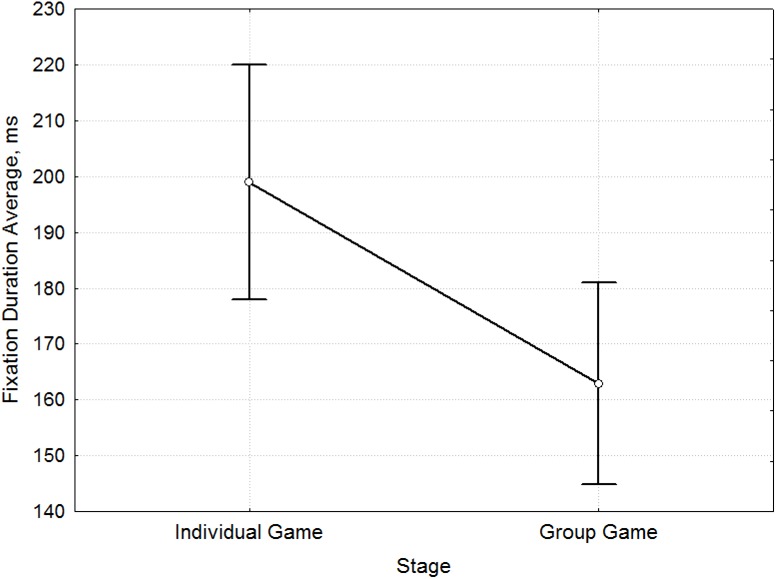
Mean comparison of Fixation Duration Average for the stages before and after Socialization. The differences in Fixation Duration Average [ms] (y-axis) between the Individual Game and Group Game stages (x-axis).

During the Individual Game stage, the participants demonstrated lower fixation frequency (Fig 7) (3.11 fixations per second before Socialization to 5.76 fixations per second after Socialization; F = 11.02, p = 0.001, S7 Table), but longer fixation duration (Fig 8) (201.21 ms before and 162.86 ms after Socialization; F = 7.52, p = 0.007, S8 Table) compared to the Group Game stage. Thus, the decision making process after socialization is accompanied by an increase in the frequency of fixation and reduction of their duration.

**Fig 9:**
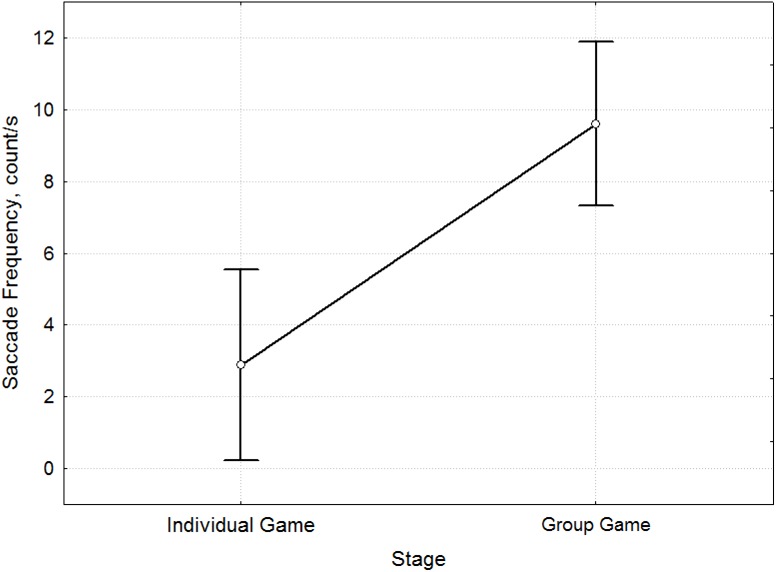
Mean comparison of Saccade Frequency for the stages before and after socialization. The differences in Saccade Frequency [count/s] (y-axis) between the Individual Game and Group Game stages (x-axis).

**Fig 10:**
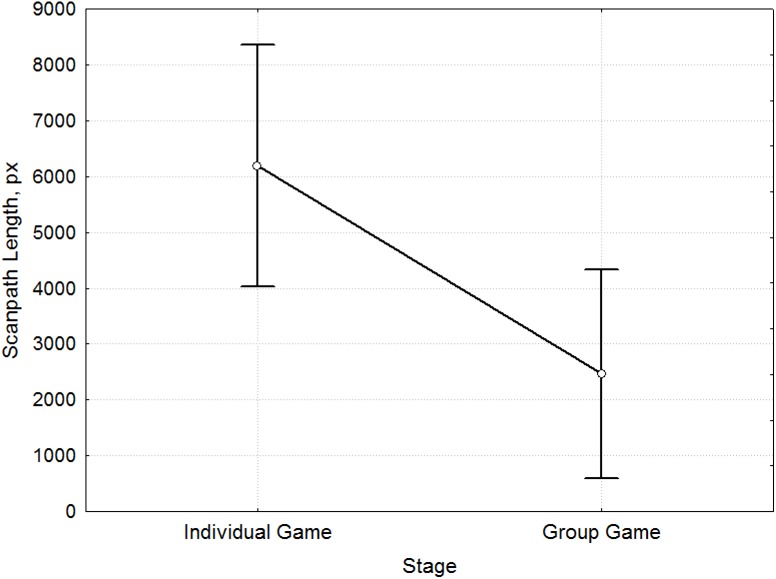
Mean comparison of Scanpath Length for the stages before and after Socialization. The differences in Scanpath Length [px] (y-axis) between Individual Game and Group Game stages (x-axis).

Comparison of the characteristics of the saccades (Fig 9) also revealed differences between the stages before and after Socialization (F = 13.98, p <0.001, S9 Table). During the Individual Game, a lower frequency of eye movement was observed. Scanpath Length (Fig 10) was greater during this stage (F = 6.8, p = 0.01, S10 Table). This was due to the duration of decision making before the choice of possible answers (an average length of 12553.5 ms in Individual Game and 4857.4 ms in Group Game).

Thus, the decision making process after Socialization is characterized by greater Saccade Frequency and smaller Scanpath Length. The results can be summed up as follows: The process of payoff matrix analysis before Socialization is characterized by a higher concentration of attention by participants, which is expressed by their considering the options offered to them for a longer time. During the Group Game stage (after Socialization), the analysis of the Prisoner's Dilemma was faster. As we compared eye movements during different stages, we were inclined to believe that these results were because the participants saw each round as a new task during the Individual Game stage. After Socialization, they acquired certain ideas that they followed, and particular strategies were formed and used by participants, which led to a change in the nature of their perception of conditions in the subsequent rounds.

This conclusion is confirmed by the participants’ self-reports. Four of nine participants with ETG said that after Socialization they tried to behave more cooperatively, that is, they tried to use a cooperative strategy.

## Conclusions

We used eye-tracking to study and forecast the decision making process in social interaction in the Prisoners’ Dilemma game. Results showed that during the Individual Game stage, participants explore the payoff matrix in every new trial and so every trial is perceived as a new challenge. During the Group Game stage, participants perceive the rounds as a series and spend less time making decisions. Their analysis of the payoff matrix at the stage of the Group Game is more thorough: The decision making process is accompanied by an increase in Fixation Frequency and reduction of Fixation Duration, greater Saccade Frequency, and a smaller Scanpath Length. This analysis of the payoff matrix is in accordance with a particular strategy formed under the influence of sociality on the basis of group identity.

Socialization promotes group identity development through interaction with other and affects cooperation during the Group Game stage. Introduction of the Socialization stage allows tracking changes in the characteristics of oculomotor activity in areas corresponding to choices to cooperate or defect, depending on the level of cooperation and its dynamics. Thus, the increase in Dwell Time and Fixation Time for noncooperative gains is associated with a preference for noncooperative choices and a decline in the overall level of cooperation.

A greater number of fixations on the group name corresponds to a strong group identity, but it is not related to a decision defect or cooperate.

This study demonstrates that eye-tracking systems can be used in group experiments. We suggest further development of eye-tracking study of the influence of social factors on decision making, to expand the subject matter from the visual perception of elements of the payoff matrix to the attention paid by participants to other players. The ability to manipulate the size of the room, the location of the computers, and other aspects of the physical space will enable closer investigation of the effect of social factors on cooperation and the effect of the organization of space on decision making.

## Supporting information

S1 Table Participant’s choices during Individual Game (before Socialization stage) in the Prisoner’s Dilemma game. c–cooperative choice, n–noncooperative choice.
(DOCX)

S2 Table Participant’s choices during Group Game (after Socialization stage) in the Prisoner’s Dilemma game. c–cooperative choice, n–noncooperative choice.
(DOCX)

S3 Table Differences in Dwell Time (%) for AOI 4. The differences in Dwell Time between Cooperators and Defectors according to the behavior data from the Prisoners’ Dilemma game during the Individual Game and Group Game stages.
(DOCX)

S4 Table Differences in Fixation Time (%) for AOI 4. The differences in Fixation Time between Cooperators and Defectors according to the behavior data from the Prisoners’ Dilemma game during the Individual Game and Group Game stages.
(DOCX)

S5 Table Participant’s index of group identity measured on the Fishbach-Ellemers scale. Each participant evaluated his or her relationship with the group formed at the Socialization stage from 1 (weak) to 7 (strong) points. 4.5 points were taken as a border result, dividing the participants with a weak and a strong group identity.
(DOCX)

S6 Table Differences in Fixation Frequency on group name in participants with a weak and strong group identity. The differences in Fixation Count between participants with weak and strong group identity.
(DOCX)

S7 Table Mean comparison of Fixation Frequency for the stages before and after socialization. The differences in Fixation Frequency [count/s] between the Individual Game and Group Game stages.
(DOCX)

S8 Table Mean comparison of Fixation Duration Average for the stages before and after socialization. The differences in Fixation Duration Average [ms] between the Individual Game and Group Game stages.
(DOCX)

S9 Table Mean comparison of Saccade Frequency for the stages before and after socialization. The differences in Saccade Frequency [count/s] between the Individual Game and Group Game stages.
(DOCX)

S10 Table Mean comparison of Scanpath Length for the stages before and after socialization. The differences in Scanpath Length [px] between Individual Game and Group Game stages.
(DOCX)

## References

[1] Pfeiffer UJ, Vogeley K, Schilbach L. From gaze cueing to dual eye-tracking: novel approaches to investigate the neural correlates of gaze in social interaction. Neuroscience & Biobehavioral Reviews. 2013;37: 2516–2528. 23928088 10.1016/j.neubiorev.2013.07.017

[2] Xia C-Y, Meloni S, Perc M, Moreno Y. Dynamic instability of cooperation due to diverse activity patterns in evolutionary social dilemmas. EPL (Europhysics letters). 2015;109: 58002.

[3] Chen M, Wang L, Sun S, Wang J, Xia C. Evolution of cooperation in the spatial public goods game with adaptive reputation assortment. Physics Letters A. 2016;380: 40–47.

[4] Wang J, Lu W, Liu L, Li L, Xia C. Utility Evaluation Based on One-To-N Mapping in the Prisoner’s Dilemma Game for Interdependent Networks. PloS one. 2016;11: e0167083 doi: 10.1371/journal.pone.0167083 27907024 10.1371/journal.pone.0167083 PMC5131937

[5] Sun S, Li R, Wang L, Xia C. Reduced synchronizability of dynamical scale-free networks with onion-like topologies. Applied Mathematics and Computation. 2015;252: 249–256.

[6] Sun S, Wu Y, Ma Y, Wang L, Gao Z, Xia C. Impact of Degree Heterogeneity on Attack Vulnerability of Interdependent Networks. Scientific Reports. 2016;6: 32983 doi: 10.1038/srep32983 27609483 10.1038/srep32983 PMC5016735

[7] Sun S, Ma Y, Wu Y, Wang L, Xia C. Towards structural controllability of local-world networks. Physics Letters A. 2016;380: 1912–1917.

[8] Meng X-K, Xia C-Y, Gao Z-K, Wang L, Sun S-W. Spatial prisoner’s dilemma games with increasing neighborhood size and individual diversity on two interdependent lattices. Physics Letters A. 2015;379: 767–773.

[9] Xia C-Y, Meng X-K, Wang Z. Heterogeneous Coupling between Interdependent Lattices Promotes the Cooperation in the Prisoner’s Dilemma Game. PLOS ONE. 2015;10: e0129542 doi: 10.1371/journal.pone.0129542 26102082 10.1371/journal.pone.0129542 PMC4477883

[10] Arieli A, Ben-Ami Y, Rubinstein A. Tracking decision makers under uncertaint. American Economic Journal: Microeconomics. 2011;3: 68–76.

[11] Devetag G, Di Guida S, Polonio L. An eye-tracking study of feature-based choice in one-shot games. Experimental Economics. 2016;19: 177–201.

[12] Knoepfle DT, Wang JT, Camerer CF. Studying Learning in Games Using Eye‐tracking. Journal of the European Economic Association. 2009;7: 388–398.

[13] Krajbich I, Armel C, Rangel A. Visual fixations and the computation and comparison of value in simple choice. Nature neuroscience. 2010;13: 1292–1298. doi: 10.1038/nn.2635 20835253 10.1038/nn.2635

[14] Stewart N, Gächter S, Noguchi T, Mullett TL. Eye movements in strategic choice. Journal of Behavioral Decision Making. 2016; 29: 137–156. doi: 10.1002/bdm.1901 27513881 10.1002/bdm.1901 PMC4959529

[15] Ettinger U, Kumari V, Crawford TJ, Davis RE, Sharma T, Corr PJ. Reliability of smooth pursuit, fixation, and saccadic eye movements. Psychophysiology. 2003;40: 620–628. 14570169 10.1111/1469-8986.00063

[16] Nagel R. Unraveling in guessing games: An experimental study. The American Economic Review. 1995;85: 1313–1326.

[17] Stahl DO, Wilson PW. Experimental evidence on players’ models of other players. Journal of economic behavior & organization. 1994;25: 309–327.

[18] Stahl DO, Wilson PW. On players′ models of other players: Theory and experimental evidence. Games and Economic Behavior. 1995;10: 218–254.

[19] Camerer CF, Ho T-H, Chong J-K. A cognitive hierarchy model of games. The Quarterly Journal of Economics. 2004;119: 861–898.

[20] Ho T-H, Camerer C, Weigelt K. Iterated dominance and iterated best response in experimental”p-beauty contests.” The American Economic Review. 1998;88: 947–969.

[21] Rapoport A, Chammah AM. Prisoner’s dilemma: A study in conflict and cooperation. University of Michigan press; 1965.

[22] Andreoni J, Miller JH. Rational cooperation in the finitely repeated prisoner’s dilemma: Experimental evidence. The economic journal. 1993;103: 570–585.

[23] Kreps DM, Milgrom P, Roberts J, Wilson R. Rational cooperation in the finitely repeated prisoners’ dilemma. Journal of Economic theory. 1982;27: 245–252.

[24] Ellison G. Cooperation in the prisoner’s dilemma with anonymous random matching. The Review of Economic Studies. 1994;61: 567–588.

[25] Aronson E. The social animal 5 th ed. NYWH Freeman 1988.

[26] Bowlby J. Attachment and loss: Retrospect and prospect. American journal of Orthopsychiatry. 1982;52: 664–678. 7148988 10.1111/j.1939-0025.1982.tb01456.x

[27] Neugebauer T, Perote J, Schmidt U, Loos M. Selfish-biased conditional cooperation: On the decline of contributions in repeated public goods experiments. Journal of Economic Psychology. 2009;30: 52–60.

[28] Fischbacher U, Gächter S, Fehr E. Are people conditionally cooperative? Evidence from a public goods experiment. Economics letters. 2001;71: 397–404.

[29] Grujić J, Fosco C, Araujo L, Cuesta JA, Sánchez A. Social Experiments in the Mesoscale: Humans Playing a Spatial Prisoner’s Dilemma. PLOS ONE. 2010;5: e13749 doi: 10.1371/journal.pone.0013749 21103058 10.1371/journal.pone.0013749 PMC2980480

[30] Nowak MA, Sigmund K. Evolution of indirect reciprocity by image scoring. Nature. 1998;393: 573–577. doi: 10.1038/31225 9634232 10.1038/31225

[31] Riolo RL, Cohen MD, Axelrod R. Evolution of cooperation without reciprocity. Nature. 2001;414: 441–443. doi: 10.1038/35106555 11719803 10.1038/35106555

[32] Dasgupta N. Implicit ingroup favoritism, outgroup favoritism, and their behavioral manifestations. Social Justice Research. 2004;17: 143–169.

[33] Tajfel H, Billig MG, Bundy RP, Flament C. Social categorization and intergroup behaviour. European journal of social psychology. 1971;1: 149–178.

[34] Berkman ET, Lukinova E, Menshikov I, Myagkov M. Sociality as a Natural Mechanism of Public Goods Provision. PLOS ONE. 2015;10: e0119685 doi: 10.1371/journal.pone.0119685 25790099 10.1371/journal.pone.0119685 PMC4366235

[35] Lukinova E, Myagkov M, Shishkin P. The value of sociality. Foresight. 2014;16: 309–328.

[36] Tajfel H, Turner JC. An integrative theory of intergroup conflict. The social psychology of intergroup relations. 1979;33–48.

[37] Babkina T, Myagkov M, Lukinova E, Peshkovskaya A, Menshikova O, Berkman ET. Choice of the group increases intra-cooperation. CEUR-Workshop. 2016;1627: 13–24. Available from: https://cla2016.hse.ru/data/2016/07/24/1119025624/EEML2016.pdf.

[38] Fischbacher U. z-Tree: Zurich toolbox for ready-made economic experiments. Experimental economics. 2007;10: 171–178.

[39] Ellemers N, Spears R, Doosje B. Sticking together or falling apart: In-group identification as a psychological determinant of group commitment versus individual mobility. Journal of personality and social psychology. 1997;72: 617–626.

