# Supplementary material for: The socialization effect on decision making in the Prisoner's Dilemma game: An eye-tracking study
Source: PLoS One. 2017 Apr 10;12(4):e0175492. doi: 10.1371/journal.pone.0175492 (PMC5386283; doi:10.1371/journal.pone.0175492)
Supplement: S2 Table — c–cooperative choice, n–noncooperative choice. (DOCX) [file pone.0175492.s002.docx]

**S2 Table. Participant’s choices during Group Game (after Socialization stage) in the Prisoner’s Dilemma game.** c – cooperative choice, n – noncooperative choice.

| **Participant** | **№1** | **№2** | **№3** | **№4** | **№5** | **№6** | **№7** | **№8** | **№9** |
| --- | --- | --- | --- | --- | --- | --- | --- | --- | --- |
| **Round** | **Choice** | **Choice** | **Choice** | **Choice** | **Choice** | **Choice** | **Choice** | **Choice** | **Choice** |
| 1 | c | n | n | n | c | c | c | c | n |
| 2 | c | n | n | c | c | c | c | c | n |
| 3 | c | n | n | c | c | c | c | c | c |
| 4 | n | n | c | n | c | c | c | c | n |
| 5 | c | n | n | c | n | c | c | n | c |
| 6 | n | n | n | n | c | c | c | c | n |
| 7 | c | n | c | c | c | c | c | c | c |
| 8 | n | n | c | n | c | c | c | c | n |
| 9 | c | n | n | n | c | c | c | c | n |
| 10 | c | n | c | n | n | c | c | n | n |
| 11 | c | n | n | n | c | c | c | c | n |
| 12 | c | n | n | n | n | c | c | n | n |
| 13 | c | n | n | n | n | c | c | n | c |
| 14 | c | n | n | c | c | c | c | c | c |
| 15 | c | n | n | c | n | c | c | n | c |
| **% of cooperation** | **80** | **0** | **26.7** | **40** | **66.7** | **100** | **100** | **66.7** | **40** |
