# Supplementary material for: The socialization effect on decision making in the Prisoner's Dilemma game: An eye-tracking study
Source: PLoS One. 2017 Apr 10;12(4):e0175492. doi: 10.1371/journal.pone.0175492 (PMC5386283; doi:10.1371/journal.pone.0175492)
Supplement: S3 Table — The differences in Dwell Time between Cooperators and Defectors according to the behavior data from the Prisoners’ Dilemma game during the Individual Game and Group Game stages. (DOCX) [file pone.0175492.s003.docx]

**S3 Table. Differences in Dwell Time (%) for AOI 4.** The differences in Dwell Time between Cooperators and Defectors according to the behavior data from the Prisoners’ Dilemma game during the Individual Game and Group Game stages.

| **Dwell Time (%) for AOI 4** | **Cooperators** | | **Defectors** | |
| --- | --- | --- | --- | --- |
|  | Individual Game | Group Game | Individual Game | Group Game |
| Mean | 6,62 | 2,62 | 5,55 | 9,23 |
| SD | 6,28 | 3,30 | 3,91 | 6,89 |
| Lower 95% CI | 4,58 | 1,21 | 3,64 | 5,02 |
| Upper 95% CI | 8,65 | 4,03 | 7,46 | 13,44 |
