# Supplementary material for: The socialization effect on decision making in the Prisoner's Dilemma game: An eye-tracking study
Source: PLoS One. 2017 Apr 10;12(4):e0175492. doi: 10.1371/journal.pone.0175492 (PMC5386283; doi:10.1371/journal.pone.0175492)
Supplement: S4 Table — The differences in Fixation Time between Cooperators and Defectors according to the behavior data from the Prisoners’ Dilemma game during the Individual Game and Group Game stages. (DOCX) [file pone.0175492.s004.docx]

**S4 Table. Differences in Fixation Time (%) for AOI 4.** The differences in Fixation Time between Cooperators and Defectors according to the behavior data from the Prisoners’ Dilemma game during the Individual Game and Group Game stages.

| **Fixation Time (%) for AOI 4** | **Cooperators** | | **Defectors** | |
| --- | --- | --- | --- | --- |
|  | Individual Game | Group Game | Individual Game | Group Game |
| Mean | 5,96 | 2,42 | 5,10 | 8,21 |
| SD | 5,54 | 3,57 | 2,90 | 5,75 |
| Lower 95% CI | 4,17 | 1,13 | 3,42 | 4,10 |
| Upper 95% CI | 7,76 | 3,71 | 6,78 | 12,32 |
