# Supplementary material for: The socialization effect on decision making in the Prisoner's Dilemma game: An eye-tracking study
Source: PLoS One. 2017 Apr 10;12(4):e0175492. doi: 10.1371/journal.pone.0175492 (PMC5386283; doi:10.1371/journal.pone.0175492)
Supplement: S5 Table — Each participant evaluated his or her relationship with the group formed at the Socialization stage from 1 (weak) to 7 (strong) points. 4.5 points were taken as a border result, dividing the participants with a weak and a strong group identity. (DOCX) [file pone.0175492.s005.docx]

**S5 Table. Participant’s index of group identity measured on the Fishbach-Ellemers scale.** Each participant evaluated their relationship with their group formed at the Socialization stage from 1 (weak) to 7 (strong) points. 4.5 points were taken as a border result, dividing the participants with a weak and a strong group identity.

| **Participant** | **Index of group identity** | **Weak or strong group identity** |
| --- | --- | --- |
| №1 | 6.00 | strong |
| №2 | 2.00 | weak |
| №3 | 4.67 | strong |
| №4 | 3.33 | weak |
| №5 | 5.33 | strong |
| №6 | 6.33 | strong |
| №7 | 3.67 | weak |
| №8 | 4.67 | strong |
| №9 | 3.00 | weak |
