# Supplementary material for: The socialization effect on decision making in the Prisoner's Dilemma game: An eye-tracking study
Source: PLoS One. 2017 Apr 10;12(4):e0175492. doi: 10.1371/journal.pone.0175492 (PMC5386283; doi:10.1371/journal.pone.0175492)
Supplement: S6 Table — The differences in Fixation Count between participants with weak and strong group identity. (DOCX) [file pone.0175492.s006.docx]

**S6 Table. Differences in Fixation Frequency on group name in participants with a weak and strong group identity.** The differences in Fixation Count between participants with weak and strong group identity.

| **Fixation Frequency** | **Group Identity** | |
| --- | --- | --- |
|  | **Weak** | **Strong** |
| Mean | 1,27 | 2,49 |
| SD | 1,31 | 1,70 |
| Lower 95% CI | 0,66 | 1,87 |
| Upper 95% CI | 1,89 | 3,10 |
