# Supplementary material for: The socialization effect on decision making in the Prisoner's Dilemma game: An eye-tracking study
Source: PLoS One. 2017 Apr 10;12(4):e0175492. doi: 10.1371/journal.pone.0175492 (PMC5386283; doi:10.1371/journal.pone.0175492)
Supplement: S7 Table — The differences in Fixation Frequency [count/s] between the Individual Game and Group Game stages. (DOCX) [file pone.0175492.s007.docx]

**S7 Table. Mean comparison of Fixation Frequency for the stages before and after socialization.** The differences in Fixation Frequency [count/s] between the Individual Game and Group Game stages.

| **Fixation Frequency [count/s]** | **Mean** | **SD** | **Lower 95% CI** | **Upper 95% CI** |
| --- | --- | --- | --- | --- |
| Individual Game Stage | 3,07 | 0,98 | 2,87 | 3,28 |
| Group Game Stage | 5,76 | 7,41 | 4,42 | 7,11 |
