# Supplementary material for: The socialization effect on decision making in the Prisoner's Dilemma game: An eye-tracking study
Source: PLoS One. 2017 Apr 10;12(4):e0175492. doi: 10.1371/journal.pone.0175492 (PMC5386283; doi:10.1371/journal.pone.0175492)
Supplement: S8 Table — The differences in Fixation Duration Average [ms] between the Individual Game and Group Game stages. (DOCX) [file pone.0175492.s008.docx]

**S8 Table. Mean comparison of Fixation Duration Average for the stages before and after socialization.** The differences in Fixation Duration Average [ms] between the Individual Game and Group Game stages.

| **Fixation Duration Average [ms]** | **Mean** | **SD** | **Lower 95% CI** | **Upper 95% CI** |
| --- | --- | --- | --- | --- |
| Individual Game Stage | 198,92 | 58,17 | 186,60 | 211,25 |
| Group Game Stage | 162,86 | 121,83 | 140,74 | 184,98 |
