# Supplementary material for: The socialization effect on decision making in the Prisoner's Dilemma game: An eye-tracking study
Source: PLoS One. 2017 Apr 10;12(4):e0175492. doi: 10.1371/journal.pone.0175492 (PMC5386283; doi:10.1371/journal.pone.0175492)
Supplement: S9 Table — The differences in Saccade Frequency [count/s] between the Individual Game and Group Game stages. (DOCX) [file pone.0175492.s009.docx]

**S9 Table. Mean comparison of Saccade Frequency for the stages before and after socialization.** The differences in Saccade Frequency [count/s] between the Individual Game and Group Game stages.

| **Saccade Frequency [count/s]** | **Mean** | **SD** | **Lower 95% CI** | **Upper 95% CI** |
| --- | --- | --- | --- | --- |
| Individual Game Stage | 2,88 | 0,93 | 2,68 | 3,08 |
| Group Game Stage | 9,60 | 16,64 | 6,576 | 12,62 |
