# Supplementary material for: The socialization effect on decision making in the Prisoner's Dilemma game: An eye-tracking study
Source: PLoS One. 2017 Apr 10;12(4):e0175492. doi: 10.1371/journal.pone.0175492 (PMC5386283; doi:10.1371/journal.pone.0175492)
Supplement: S10 Table — The differences in Scanpath Length [px] between Individual Game and Group Game stages. (DOCX) [file pone.0175492.s010.docx]

**S10 Table. Mean comparison of Scanpath Length for the stages before and after socialization.** The differences in Scanpath Length [px] between Individual Game and Group Game stages.

| **Scanpath Length [px]** | **Mean** | **SD** | **Lower 95% CI** | **Upper 95% CI** |
| --- | --- | --- | --- | --- |
| Individual Game Stage | 6189,01 | 15568,90 | 2890,28 | 9487,75 |
| Group Game Stage | 2461,25 | 2437,38 | 2018,79 | 2903,71 |
